# Context of Violence in Adolescence Cohort (CoVAC) study: protocol for a mixed methods longitudinal study in Uganda

**DOI:** 10.1186/s12889-019-7654-8

**Published:** 2020-01-13

**Authors:** Karen Devries, Jenny Parkes, Louise Knight, Elizabeth Allen, Sophie Namy, Simone Datzberger, Winifred Nalukenge, Lydia Atuhaire, Nambusi Kyegombe, Eddy Walakira, Janet Seeley, Helen A. Weiss, Dipak Naker

**Affiliations:** 1 London School of Hygiene and Tropical Medicine, 15-17 Tavistock Place, London, WC1H 9SH UK; 2 University College London-Institute of Education, 2 0 Bedford Way, London, WC1H 0AL UK; 3 Raising Voices, Plot 16, Tufnell Drive, Kamwokya, Kampala, Uganda; 4 Medical Research Council/Uganda Virus Research Institute and London School of Hygiene and Tropical Medicine, Uganda Research Unit, PO Box 49, Entebbe, Uganda; 5 Makerere University, Kampala, Uganda; 6 MRC Tropical Epidemiology Group, London School of Hygiene & Tropical Medicine, Keppel Street, London, WC1E 7HT UK

**Keywords:** Violence, Child abuse, Intimate partner violence, Uganda, Cohort, Longitudinal, Mixed methodology implementation science, Gender, Violence at school

## Abstract

**Background:**

Violence exposure in adolescence is associated with a range of poor health and social outcomes, including both the perpetration and experience of violence in later intimate relationships. However, there is little longitudinal evidence on how both individual and contextual characteristics encourage or interrupt these associations. We designed the Contexts of Violence in Adolescence Cohort study (CoVAC) to provide evidence on these pathways for Ugandan adolescents, with the aim of providing information to improve the design of violence prevention interventions for adolescents and young adults.

**Methods:**

CoVAC is a mixed-methods prospective cohort study with three parallel strands. Between 2014 and 2022, the study comprises three waves of quantitative survey data collection; qualitative data from five time points; and a series of workshops to facilitate direct use of emerging findings by intervention developers at Uganda-based NGO Raising Voices in their ongoing work to prevent violence. 3431 adolescents participated in a survey in 2014 when the majority were aged 11–14 years, and agreed to be re-contacted for a Wave 2 survey in 2018 (aged about 15–18 years); and again in 2021 (aged 18–21 years). 36 young people from Wave 1 survey sample will be invited to participate in longitudinal qualitative data collection. Adolescents aged 18 years and over will provide informed consent; for those under age 18 years, adolescents will be invited to assent, except in cases where caregivers, following notification, have opted not to consent to their adolescent’s participation. Quantitative and qualitative data will be analysed iteratively, and triangulation will be used to confirm, clarify and deepen our interpretation of findings. We will hold regular structured meetings so that emerging findings can be integrated into intervention development.

**Discussion:**

This will be the first longitudinal study on the aetiology of violence over adolescence in sub-Saharan Africa which will enable examination of pathways using mixed methods at multiple time points. Quantitative mediation analysis, and annual qualitative fieldwork will provide detailed insights into how adolescents’ violence-related experiences, perspectives and practices relate to their social contexts and how these change over time. Results will feed directly into intervention development to reduce violence and harmful sequelae.

**Trial registration:**

This study is a long-term follow up of participants in the Good Schools Study (NCT01678846, clinicaltrials.gov). This protocol is for cohort follow-up only; we have a separate protocol paper describing an evaluation of the long-term effects of the Good School Toolkit (In preparation).

## Background

Globally, more than 1 billion children report physical, sexual or emotional violence every year [1], and one in three adult women experience intimate partner violence in their lifetime [2]. In cross-sectional studies, exposure to physical, sexual or emotional violence during childhood and adolescence are established risk factors for experience of intimate partner and sexual violence in adult women [3], and perpetration of intimate partner and other forms of violence in men [4, 5] However, evidence from cohort studies is less clear. There have been relatively few cohort studies on the relationship between violence in childhood and adolescence and that in young adulthood, with some finding associations in women but not men [6], and some finding associations between early exposure and later perpetration (but not victimisation) in both sexes [7]. Consequently, little is known about the pathways by which early and later violence are associated, and which individual and contextual factors affect progress along, or disrupt, these pathways. Little is known also about the varying effects on these pathways of different types and levels of violence. In particular, there is a lack of evidence from low and middle income country settings, where both the patterns of early violence exposure and elements of context may differ substantially from high income settings.

### Ugandan study context

Uganda is a low income country with a population of approximately 44 million people, of whom 19.3 million (55%) are under 18 years old [8]. More than 90% of boys and girls attend at least some schooling, however, only about 50% of children enrolled complete primary school nationally, and just under 60% who have completed primary school attend secondary [9]. Uganda is ranked 126 of 160 countries on the Gender Inequality Index 2017 [10] and ‘sexual abuse and violence’ is one of the top risk factors for disease burden [11]. The country has legislation, policy and national action plans to address child maltreatment, intimate partner violence and sexual violence, although faces challenges in implementation as in other resource poor settings. The recent national Violence Against Children Survey found that 35.5% of female and 16.5% of male 18–24 year olds reported experiencing sexual violence under age 18 years [12].

The study is situated in Luwero District, Uganda. Luwero is demographically similar to Uganda as a whole, with levels of small-scale farming and electricity coverage similar to the national average. It has rural and urban areas and borders Kampala, the capital of Uganda. Our previous work indicates high levels of violence in Luwero, with more than 90% of early adolescents attending primary school reporting lifetime exposure to physical violence [13].The current study is a continuation of an existing partnership between the London School of Hygiene and Tropical Medicine (LSHTM),University College London Institute of Education (UCL Institute of Education), Raising Voices, and Makerere University; with the addition of the Uganda MRC-UVRI. We will draw on data collected in 2014 during the Good Schools Study [14–16], and will follow participants from 2018 to 2021, as they transition from early adolescence to early adulthood.

This will be a mixed methods study with quantitative and qualitative research components, with a third stream of work where emerging research results are input directly into ongoing intervention development. Specifically, we will use quantitative survey data at 3 time points and qualitative data at 5 time points to: 1) examine the epidemiology and patterns of violence exposure in early adolescence; 2) understand which patterns of early adolescent exposure to violence are associated with violence use and experience in later adolescence and young adulthood; 3) explore the pathways by which violence in early adolescence, later adolescence and young adulthood are associated and how context encourages or interrupts these associations. Our overarching *hypothesis* is that context matters in shaping whether and how early violence exposure leads to later violence use and experience. Specific aims and hypotheses are outlined below.

### Aims and hypotheses

#### Aim 1

To examine the epidemiology and patterns of violence exposure in adolescence.

Quantitative: We will document patterns of self-reported exposure to violence over time, combining information on physical, sexual and emotional violence and neglect, timing, frequency, severity and perpetrator, and the ‘normative nature’ of the violence. Hypotheses:

1.1 There will be an underlying patterning of violence exposure at waves 1, 2 and 3
1.2 The patterns/groupings observed in wave 1 will persist at wave 2 and wave 3

Qualitative: We will explore how young people understand, experience and interpret different forms and patterns of violence exposure.

#### Aim 2

(Main aim for quantitative study). To understand which patterns of early adolescent exposure to violence are associated with violence use and experience in later adolescence and young adulthood.

Quantitative: We will explore the magnitude of the association between different patterns of early adolescent violence exposure and late adolescent and early adult outcomes. Hypotheses:

2.1 The likelihood of use and experience of intimate partner violence at wave 3 will differ by wave 1 violence exposure group

Qualitative: We will explore how young people’s subjectivities, including their values, beliefs and practices, shape their responses to and are shaped by earlier experiences of different patterns of violence.

#### Aim 3

To explore the pathways by which violence in early adolescence, later adolescence and young adulthood are associated, and how context encourages or interrupts these associations.

Quantitative: We will explore whether exposure to specific individual and contextual factors mediate or moderate the relationship between early exposures and later use and experience of violence. Hypotheses:

3.1 Being male or female will moderate the association between Wave 1 violence exposure and Wave 3 intimate partner violence use and experience
3.2 Whether Wave 1 violence exposure is considered socially normative will moderate the association between Wave 1 violence exposure and Wave 3 intimate partner violence use and experience
3.3 Mental health status at Wave 2 will mediate the association between Wave 1 violence exposure and Wave 3 intimate partner violence use and experience
3.4 Having a family with no violence between caregivers and no caregiver mental health issues at Wave 2 will mediate the association between Wave 1 violence exposure and Wave 3 intimate partner violence use and experience
3.5 Being more exposed to the Good School Toolkit will mediate the association between Wave 1 violence exposure and Wave 3 intimate partner violence use and experience
3.6 Staying in school will mediate the association between Wave 1 violence exposure and Wave 3 intimate partner violence use and experience

Qualitative: We will explore how family, peer and intimate partner relationships influence young people’s responses to violence over time, including their capacity to stay safe and to sustain equitable beliefs. We will examine how school systems, practices and relationships influence girls’ and boys’ capacity to build or maintain anti-violence norms and practices over time, and how transitions to secondary school or out of school influence these processes. We will explore how community structures, norms and relationships, including religious and community justice systems, local provision of services, community organisations (e.g. women’s groups, human rights groups) and access to media (local and global) influence girls’ and boys’ perspectives and practices regarding violence. Our analysis will also explore how exposure to the language and ideas on violence prevention through the Good Schools Toolkit intervention may have influenced the ways young people subsequently frame their experiences. We will consider the role of poverty and how the political economy of the macro-level context influences these processes.

### Summary of exposures, outcomes and contexts in CoVAC

**Fig. 1:**
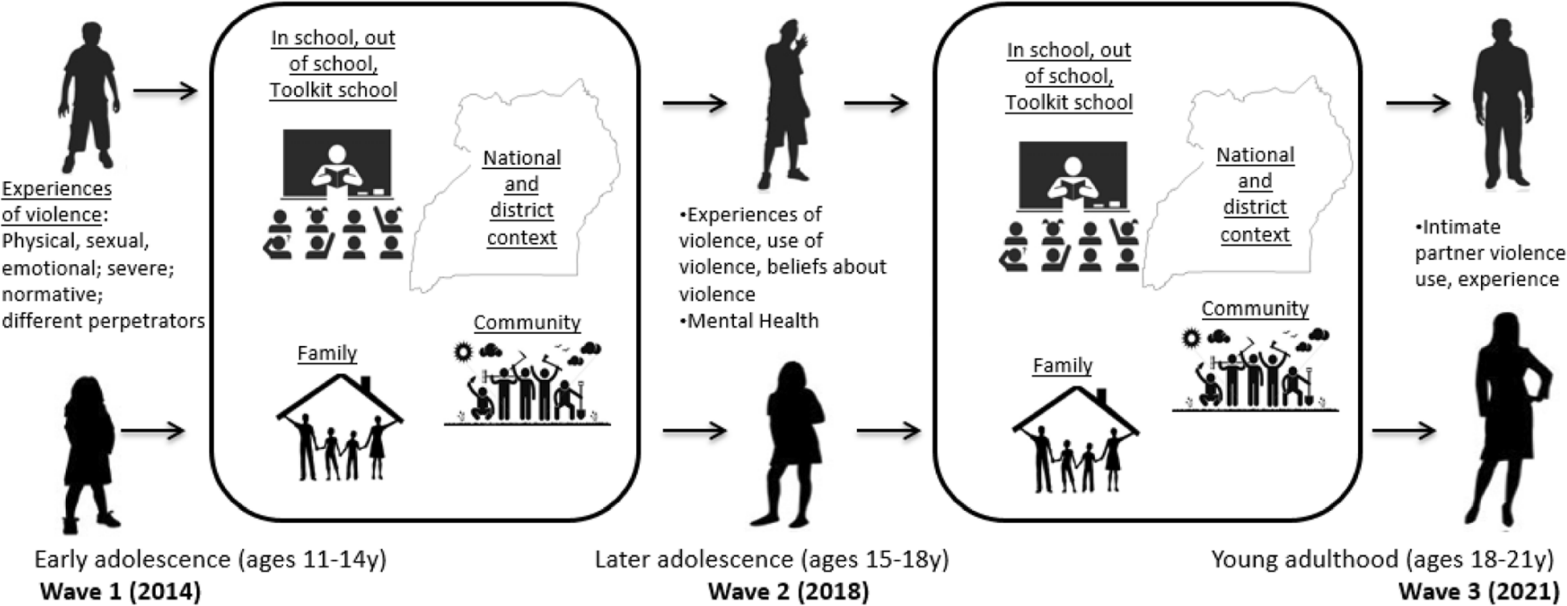
Summary of exposures, outcomes and contexts in CoVAC

For the quantitative component, the main early adolescent experiences of violence we are interested in include physical, sexual and emotional violence and neglect from school staff, peers, intimate partners, parents, other relatives, employers and any other perpetrators, as well as social perceptions of violence as normal or extreme. The main late adolescent and young adulthood outcomes of interest are use and experience of violence in intimate partnerships. The main individual and contextual factors of interest which may influence pathways are: being male or female, mental health, and family and school contexts, including whether level of exposure to the Toolkit violence prevention intervention influence later risk of violence. We will also explore the ways in which pathways are influenced by peers, community, district and national contexts (Fig. 1).

**Fig. 2:**
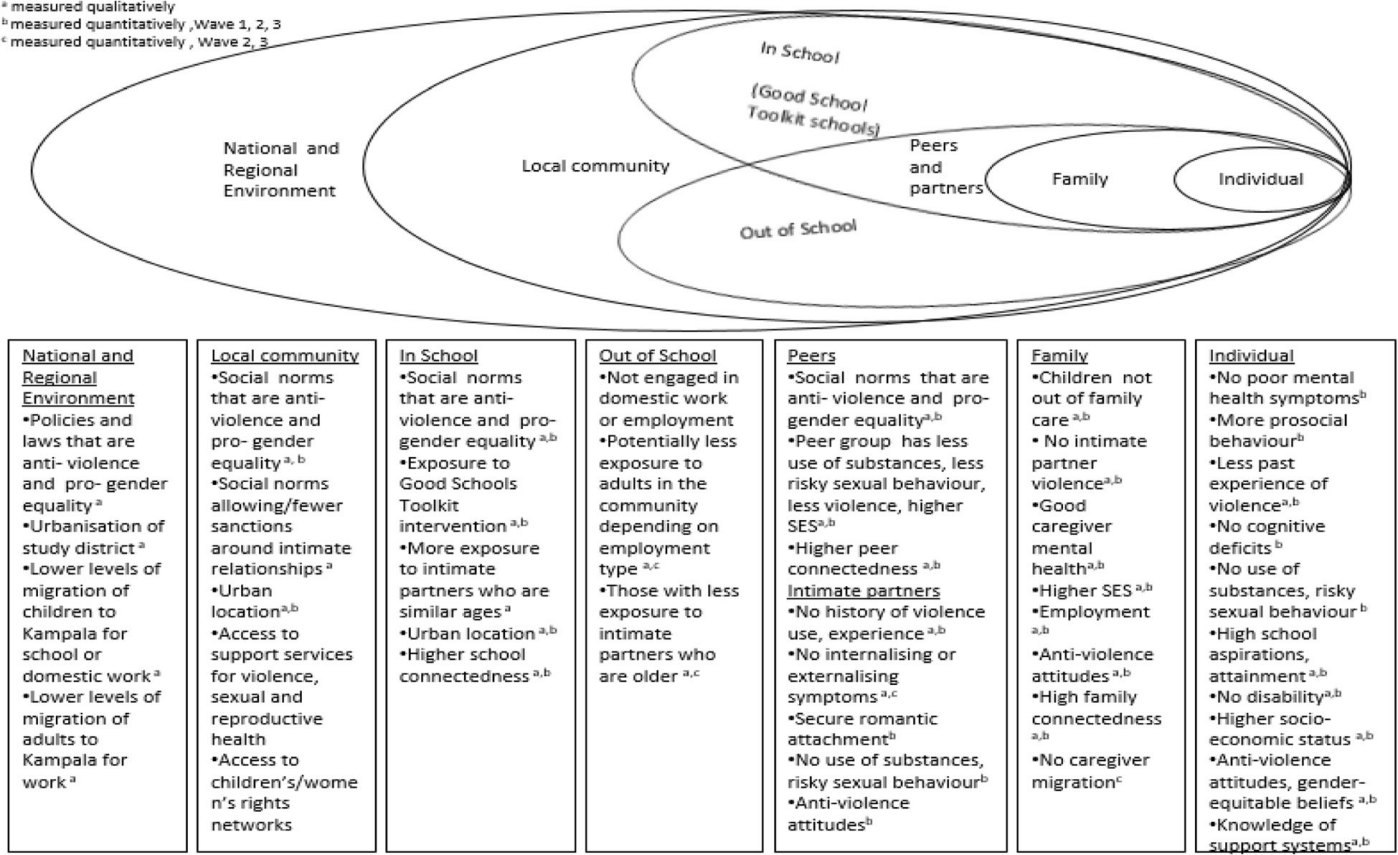
Eco-social theoretical framing, with protective factors against later violence use and experience, explored in our study

In the qualitative study, we will progressively build an explanatory framework for how structures, norms, institutions and interactions influence young people’s exposure to violence, and capacity over time to stay safe and resist the harmful effects of violence. The qualitative component is of key importance for enabling us to understand the dynamics of the relationships identified by the quantitative component. The mixed methodology will also enable the robustness of qualitative findings to be strengthened through the quantitative analysis. Central to our theoretical framing is an understanding of violence as deeply embedded within social contexts (Fig. 2) [17, 18]. Individual experiences of violence are shaped by a constellation of factors, including the surrounding norms and cultures, and resources and structural features of the contexts in which young people are situated. In order to elaborate this relationship we draw on multi-dimensional conceptualisations of violence [17], ecosocial framings of adolescents as dynamic beings shaped by and shaping layers of social context [18]. We refer not just to acts of physical, sexual and emotional force, but to the everyday interactions and identities that surround these acts, and to their roots in inequitable norms, structures and institutions [17, 19, 20]. We will build on theoretical work on the relationship between subjectivity and context, including Bourdieu’s concepts of habitus and symbolic violence [21], and positioning theory [22], which have proved fruitful in our earlier research on young people’s engagements with violence [23, 24]. These framings view adolescents as dynamic beings, positioned by and actively negotiating subject positions in relation to social contexts. We are interested in how forms of violence impact on these negotiations, and how contextual changes influence these dynamics.

## Methods

**Fig. 3:**
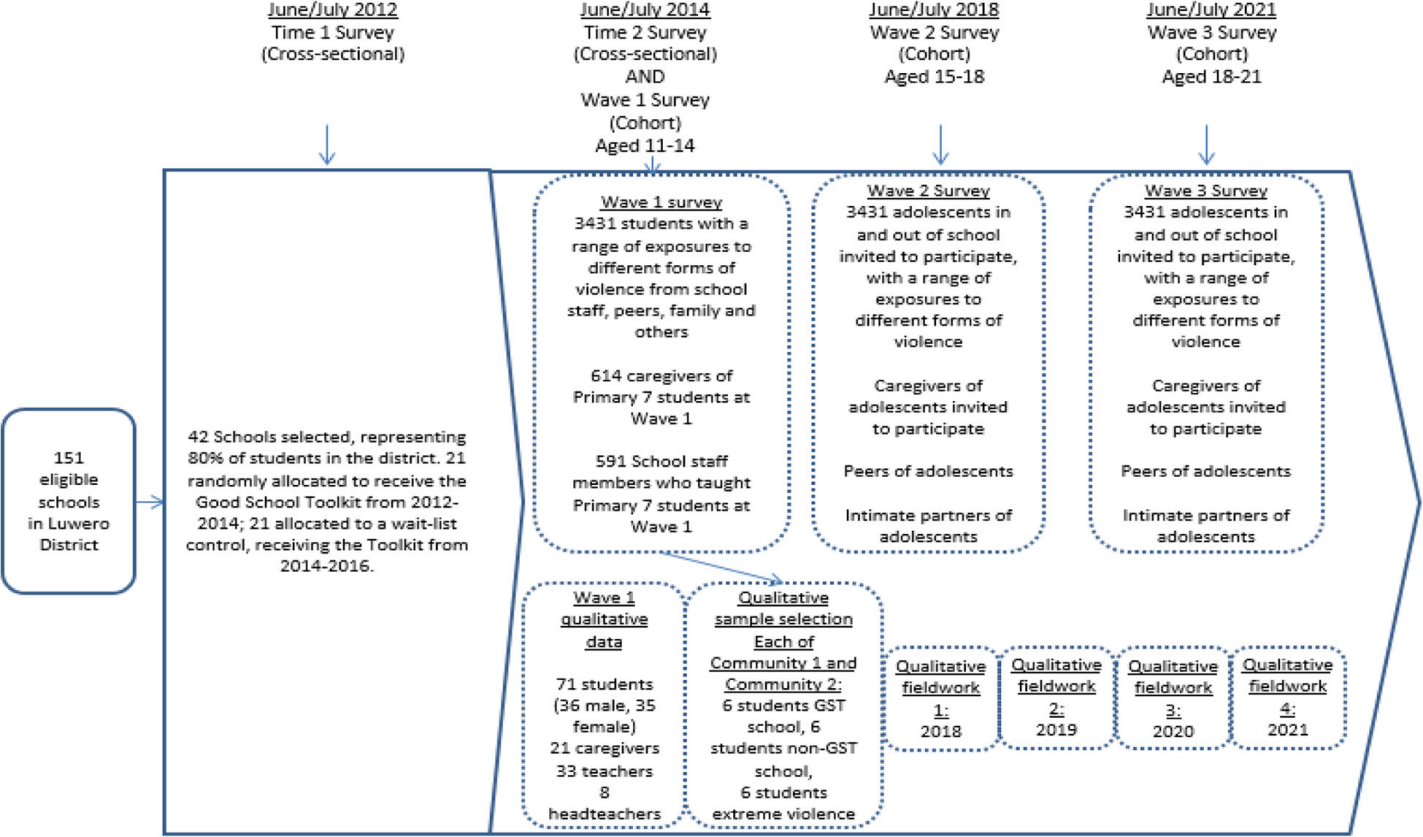
Summary of data collection over time

The research will use quantitative data collected in 2014 for the Good Schools Study (Wave 1 survey) as the baseline for a prospective cohort, with follow-up surveys in 2018 (Wave 2) and 2021 (Wave 3). The Wave 1 survey data will also be used to select participants for the qualitative research, who will be followed at 4 additional time points (Fig. 3).

### Wave 2 inclusion criteria

#### Quantitative study

Of the 3820 pupils recruited at Wave 1, 3431 (90%) agreed to be re-contacted. These pupils are the eligible sample for Wave 2 and Wave 3. Participants must be deemed able to understand survey consent procedures in English or Luganda by interviewers, so that they can give voluntary informed consent. These adolescents will be our ‘index’ participants. Other participants will be sampled in connection with the index participant. A subsample of peers, intimate partners, and caregivers identified by each index participant will also be invited to participate in a face to face survey (n = approximately 200 of each in total).

#### Qualitative study

36 girls and boys, aged 15–17 at Wave 2, will constitute the ‘core’ participants. These participants will be a subset of the index participants, and will be purposively selected based on their responses to the Wave 1 survey. The sample will include those from both rural and urban neighbourhoods, equal numbers of girls and boys, and those who have experienced more severe and less severe forms of violence. Data will also be collected from a broader sample of approximately 70 young people, 20 school staff, 30 caregivers, 20 members of communities, including religious leaders, local women’s and children’s rights organisations, police, health workers and district education officials; and from approximately 5 national level policy makers. Some of these will be invited to participate following guidance from the core participants, drawn from their social networks, including friends, intimate partners, parents/family members and teachers. Others will be selected to provide additional data on the contextual dynamics that perpetuate, prevent and protect from violence.

### Community sensitisation and cohort tracing

We plan to convene the following stakeholder groups to collaborate on the development of the research design: adolescents (in and out of school), caregivers (male and female), community leaders (local chairpersons, religious leaders, teachers, and other relevant stakeholders). We will seek feedback on study procedures, measures, how to most effectively trace and maintain contact with participants, and on communication with participants and stakeholders.

To facilitate location of individual participants, at Wave 1, we collected names, addresses, physical descriptions of house locations, mobile phone numbers of adolescents and their caregivers. We conducted small pilot follow-up exercises in 2015 and 2017, and traced 91% (*n* = 77) and traced locations of 98% (*n* = 53) in and out-of-school respectively. Our index participants will be aged about 15–18 years at the time of Wave 2 follow-up, and our core participants 15–17 years, and based on pilot tracing work we have done, will be found mainly in schools. Some adolescents will be found outside of school at various locations, including at home with their marital partners or caregivers, in domestic work, and in other types of work.

We will sensitize a variety of key stakeholders to the study and will obtain a formal letter of support from the Ministry of Education and Sports and will introduce the study in an information meeting. We will invite school headteachers from primary schools in our original trial, local education officials and any other stakeholders deemed important.

The information meeting will be facilitated by Raising Voices staff. We will develop standardised information sheets and spoken scripts to explain the study. Following ethical guidance about research on violence from WHO, and our own past research in the Good Schools Study, these scripts will not focus explicitly on violence, but will emphasise that we are tracing all participants who attended one of the 42 schools in the Good Schools Study. On the advice of our partner Raising Voices, we will refer to our survey as the ‘Footprints’ Survey, and mobile phone and qualitative research activities will also use this name in communication with community level stakeholders and individual participants. This will be in order to protect participants from potential harm related to others assuming that they have been selected to participate because they have disclosed violence.

After this initial meeting, information on the current whereabouts of the index adolescents will initially be sought from head teachers, teachers and students from the schools in the original trial. This process will yield a list of primary and secondary schools currently attended by index adolescents (as well as a number whose whereabouts are not known or who are out of school). Head teachers of schools where the index adolescents currently attend, will be contacted by phone or in person and given information about the study and which adolescents we are tracing. Those who are out of school will be traced via contacts at their last school, and by involving index adolescent and local leaders as appropriate.

We will hold a second set of information meetings with local chairpersons (who are community leaders at the local level), as well as representatives from relevant parent associations closer to the time of the Wave 2 survey. This will be facilitated in a similar way and will familiarise the broader community with the study. We will hold follow-up meetings on an occasional basis throughout the study, to maintain contact with these local representatives.

To locate out-of-school adolescents, we will approach them using contact information provided at the Wave 1 survey. Our pilot tracing exercise also indicates that their former classmates who are in secondary school also often have contact and location information for these participants. In cases where we are unable to find participants using these channels, we will also engage local chairpersons.

We intend to trace participants both within and outside Luwero District. We anticipate that a number of adolescents may have moved to Kampala (where the main research office is based), and some others may be in different districts. We have budgeted additional funds to follow-up these participants.

### Consent for index and core participants

For participants aged 18 years and over; emancipated minors; and adolescents who indicated at Wave 1 that we should not approach them at home for further contact, we will seek written informed consent for participation in research activities and to maintain contact over the study period. Emancipated minors are defined by the UNCST as “individuals below the age of majority who are pregnant, married, have a child or cater for their own livelihood.” [25] At the level of individual consent to participation, we will make clear that we are asking about ‘whether anyone has hurt’ our participants, as we have in our past Good Schools Study.

For participants who are under aged 18 years and are not emancipated minors or who indicated we can contact them at home, we will notify caregivers about the study and caregivers will be able to opt adolescents out. Adolescents, whose caregivers have not opted them out of the study, will then be approached to assent for participation prior to involvement in the study.

We will make repeat visits to ensure inclusion of as many adolescents as possible. We anticipate this will be up to 3 visits. Once index adolescents have been located, study information will be given to the adolescent to read, and will be read out loud and explained by the interviewer. The adolescent will be given time to ask questions and consider enrolment in the cohort and participation in the Wave 2 survey interview. It will be explained that agreeing to cohort enrolment means we would like to document their updated contact details and keep in touch with them over the next four years, and then interview them again in 2021.

During the consent procedure it will be clearly explained that to adolescents they do not have to participate, there are no negative consequences for choosing not partake, they can stop participating at any time during the survey or over the next four years, and that they can freely skip any question they do not want to answer during the interview. It will be made clear that consent is an ongoing process and they will be re-consented prior to Wave 3 survey. The process to withdraw participation, at any time, will be explained. No research discussion or interview will proceed without adolescents’ full voluntary informed written consent or assent. Index participants will sign a consent or assent form (or for illiterate participants, a thumbprint witnessed by a third party not involved in the study).

Participants will be provided with a laminated personal identification (ID) card. All contact information will be kept separately from survey responses– which will only be linked by a unique study ID number accessible to authorised study team members.

Those participants selected also for the qualitative study will be asked by the survey team for their consent to be approached for additional research activities. If they agree, one of the qualitative researchers will visit them at a venue of their choice, and seek their consent, clearly explaining that this will involve multiple points of contact over 1–2 months each year. They will be asked to sign an additional consent form. Written consent for participation in the qualitative study will be sought at yearly intervals; oral re-consent will be sought for any additional meetings within each year.

#### Interviewer training

We will collaborate with interviewers from our previous research as far as possible, who all speak Luganda and have direct experience working on violence research and with children in Luwero. For both the quantitative and qualitative components, interviewers will attend three weeks training on quantitative/qualitative research methods (as appropriate), ethics and consent procedures, including interview techniques such as how to listen non-judgementally, how to create and maintain rapport, and what to do if an adult or child discloses violence. During the fieldwork they will be closely supervised, with short daily debriefs, weekly reflections on the research process and ethics.

For qualitative fieldwork, the team will include 5 researchers. Two senior researchers, who are experienced ethnographers, will work alongside three field ethnographers. Where possible, one researcher will act as the key link with each core participant throughout the four fieldwork periods, to establish and maintain close research relationships over the longitudinal study. The key link researcher for all female participants will be female. During the fieldwork they will meet each day, to debrief on the day, and reflect on the research process and ethics. Work will be closely supervised, including fieldwork diaries and transcriptions.

#### Interviews

Participant preference for comfort and safety and maintaining confidentiality will be our guiding principle. Interviews will take place where they cannot be overheard and the interviewer will confirm that the participant is happy to proceed at the time and location before commencing the interview. If the situation changes, people move nearby, or the interviewee appears uncomfortable then the interviewer will pause, stop, or rearrange the interview as necessary. Participants will be informed during the consent procedure, and at the end of the interview, that if they feel they would like to talk to a counsellor about something that has come up during the interview then we can refer them to local services for support.

We plan to reimburse participants for their time spent participating in the research, with additional costs given for travel. On the advice of community advisory groups, reimbursements will be a product such as a bar of soap and/or notebook. Some additional qualitative activities, such as walking through communities and other participant-observation, will not usually require reimbursement.

### Strategies to minimise attrition between waves 2 and 3

At Wave 2 contact, for those who consent to cohort enrolment, we will record a set of detailed contact information. This will include: GPS location of the participants’ residence if they interviewed where they reside; mobile phone number if they have a phone or have access to a phone; social media contacts; contact mobile phone numbers and social media contacts of up to 3 caregivers, relatives, or others who are likely to be informed of the index adolescent’s whereabouts. Participants will be asked to nominate one of these as a ‘link person’.

We will maintain regular contact with participants and/or their given link person. We expect this will comprise of phone contact at least every 6 months, and every 3 months over key transition periods, when we know schooling is ending or a job is beginning. We will provide a toll free phone number that will appear on their ID card and encouragement to call and let us know if they are moving or their contact information is changing. We are also exploring the use of social media to facilitate ongoing contact, and will seek advice from our collaborator groups (for example, we may set up a study Facebook page if collaborators feel this would be useful and privacy can be maintained).

For those participants who are additionally engaged in the qualitative component, phone contact at least every 6 months, and every 3 months over key transition periods will supplement the multiple contacts during annual data collection periods.

### Measures for the quantitative survey

Measures in the Wave 2 survey will be a combination of measures used in our Wave 1 survey with 11–14 year olds, a further survey we have more recently conducted with 500 adolescents attending secondary school in Kampala as part of the Good School Toolkit secondary school adaptation research, and measures adapted from existing studies, which we will pre-test. In some cases we will develop simple sets of items and pre-test these. The final Wave 2 questionnaire will be pilot tested for overall length and flow, and the number of questions asked will be reduced such that overall completion time is not more than approximately one hour.

Measures are mainly from internationally recognised tools, and include: background socio-demographic information, disability (using the Washington Group Short Set of questions [26]), family and peer and school connectedness (from the Minnesota Student survey and Add Health [27]), adolescent risk and sexual behaviours (from Global School Health Surveys, the Health Behaviour in School Aged Children Surveys, National Survey of Sexual Attitudes and Lifestyles), including alcohol use (sub-set of questions from AUDIT [28]) and exposure to the Good School Toolkit intervention (developed from Good School Study [29]), previous disclosures of violence (from Good School Study), questions about paid and unpaid work (from the Good Schools Study). A summary of the main violence and mental health outcome measures are provided in Table 1.

**Table 1:** Summary of main violence and mental health outcome measures

Area	Source of questions
Violence from school staff against students	Adapted from International Society for the Prevention of Child Abuse and Neglect-Child Abuse Screening Tool-Child Institutional, ICAST-CI [30]
Violence experience and use between adolescent peers	Adapted from ICAST-CI [30]
Violence from caregivers against adolescents’	Adapted from ICAST-CI [30]
Violence from work employers against adolescents’	Adapted from ICAST-CI [30]
Adolescent Intimate Partner Violence (IPV) experience and use	Adapted from the WHO Multi-Country study on women’s health and domestic violence against women [31], and the CADRI-Conflict in Adolescent Dating Relationships Inventory [32, 33]
Student attitudes towards teacher violence	Good Schools Study [34]
Adolescents attitudes towards gender equality and violence	Gender Equitable Men scale, Uganda version [35]
Mental health measures	Strengths and Difficulties Questionnaire (SDQ-25) [36, 37] Patient Health Questionnaire-Adolescent (PHQ-A) [38]

### Qualitative research instruments

Our ethnographic, participatory research design involves flexibility in the research methods, with some methods developed through negotiation with young people themselves, in order to ensure that the design is contextually relevant and sensitive to their concerns. Data will be collected through informal observations, with the researchers spending extended time in the communities, and gathering information through informal, unstructured ‘interview-conversations’ with the core participants, often together with their peers. Field diaries, completed on a daily basis, will record these. With core participants we will conduct semi-structured biographic narrative interviews, using a topic guide. The topic guide may be modified during researcher training and fieldwork team meetings, as further themes emerge as relevant for inclusion, and so that the research team can refine and practise sensitive ways to elicit personal data (for example, about violence and dating relationships). Topic guides will also be used to facilitate interviews and focus groups with parents, peers and other adults. We will also explore a range of participatory methods, including engaging young people as co-researchers, arts/drama based work, often together with peers, and photovoice. These have proved effective in creating a relaxed atmosphere for discussing sensitive topics with children and adolescents, in helping to avoid research fatigue, and eroding power imbalances in the research relationship. These approaches will be discussed and agreed with young people. We will also consult with our partners at Raising Voices, who have expertise in working with young people in this context, as well as with our youth advisors, to ensure the qualitative methods and instruments have contextual relevance, sensitivity and appropriateness for engaging young people in Luwero.

### Statistical power

We are powered to test our main hypothesis, 2.1, whether Wave 1 levels of exposure to violence (as an example, with 25% exposed to severe violence) are associated with Wave 3 past year experience of physical, sexual and/or emotional intimate partner violence. A conservative sample size of 2406 participants (allowing for 30% loss to follow up over the study) would give us 80% power at a 5% level of significance to detect an effect difference of 7–8% between exposed and non-exposed adolescents. This assumes that 70% will have had a relationship by Wave 3 and 25% of those unexposed to severe violence at Wave 1 had past year intimate partner violence at Wave 3, which would be expected based on 2012 Uganda DHS data about relationship prevalence and intimate partner violence [39]. Analyses involving mediators and moderators are exploratory and hypothesis generating. Power calculations for these depend on a large number of factors, most of which, as is common, are unknown at this stage of the study. Fritz and MacKinnon show that a sample size of 667 provides at least 80% power to detect mediation under a range of scenarios and different tests [40]. The 7–8% difference (equivalent to an odds ratio of about 1.3 in our sample) in adult IPV prevalence between those exposed and unexposed to childhood violence that we are powered to detect is in the range of what other studies have found, for example, Widom et al. [2] show an odds ratio of 1.6. However, we have been more conservative as we expect a slightly smaller difference between groups, because baseline levels of violence exposure are higher in our study.

### Quantitative data analysis

Descriptive analysis will be conducted at each Wave. We will use latent class analysis to explore patterns of violence at Waves 1,2 and 3 and regression analysis to subsequently explore whether the patterns/groupings persist over time. Mixed regression models, allowing for clustering by school will be used to explore whether experience of intimate partner violence at Wave 3 differs by Wave 1 and Wave 2 violence exposure group. The models will include the exposure measured at both Wave 1 and 2 where available and will be adjusted for pre-specified potential confounders. Finally, further regression analysis and structural equation models will be used to explore whether individual and contextual factors mediate or moderate the relationship between early exposures and later use and experience of violence. All analysis will include information on degree to which the participant was exposed to the Good School Toolkit intervention.

### Qualitative data analysis

Analysis will begin following Fieldwork 1, so that there is an iterative process in which further data collection at Fieldwork 2, 3 and 4, are informed by the developing analysis. Management, thematic coding and searching of the transcribed personal interviews, group discussions and fieldwork diaries will be facilitated by use of NVivo. The analysis will progressively build an explanatory framework on how structures, norms, institutions and interactions influence young people’s capacity over time to resist the harmful effects of violence. Biographical case studies for each core participant will focus on their concerns about risk, safety and violence, their experiences of direct and structural violence, and how their subjectivities, including their values, beliefs and practices, shape their responses to violence, and are shaped by earlier experiences of different patterns of violence, and changes over time through late adolescence. The contextual analysis will explore how family, peer and intimate partner relationships, schools and forms of labour influence their life paths, including their capacity to build and maintain anti-violence norms and practices over time. It will investigate community structures, norms and relationships, any sustained influence of the Good Schools Toolkit, and the role of local services, social media, and broader political, legal and social structures. Selected early findings will be fed back to the participants, so that opportunities will be generated for further debate and deliberation on, for example, how social networks can support young people in staying safe and challenging violence.

### Mixing qualitative and quantitative methods

Combining our analysis of quantitative and qualitative data will strengthen the breadth and depth in our capacity to answer our research questions [41, 42]. Methodological triangulation will be used to confirm and corroborate our findings, as well as to help explain paradoxes or puzzles emerging within the qualitative or the quantitative analysis. The quantitative data will help to assess the generalisability of qualitative findings. For example, qualitative data about the perceived value of close, protective family relationships, might be corroborated by quantitative findings that higher family connectedness mediates the effects of violence. Qualitative analysis will help to interpret and clarify quantitative results; for example, the qualitative analysis of norms surrounding gender and disability could help to shed light on a quantitative association between disability and heightened exposure to physical and sexual violence. Our mixed methods analysis will be both simultaneous – for example, conducting quantitative and qualitative analyses of a selected topic following wave 2 survey and fieldwork 1 – and sequential – for example, a wave 2 survey finding of an association between alcohol/drug use in families and violence experiences could be illuminated through questions in subsequent qualitative fieldwork and analysis. Regular meetings between the qualitative and quantitative research teams, with extended discussions following analysis of each wave/fieldwork period, will be used to compare emerging findings across the two components.

### Data management

We will make use of procedures established during our Wave 1 survey. Most data collection will be in electronic format. Participant survey and consent forms will be checked against sampling lists daily. For surveys, questions are programmed to avoid missing data as a result of accidental question skips. Response inconsistencies will be checked daily using a custom Stata program. Linkage of records will be performed daily during quantitative data collection. For both the qualitative and quantitative studies, identifying data will be collected from all participants in order to enable linkage of records. This data will be stored separately in encrypted format and will only be accessible to selected senior study personnel. Child protection referrals are also made automatically based on an algorithm programmed into survey questions; and for qualitative research, manually on a case by case basis according to the same referral criteria. These are collated and cross-checked daily by the study team to ensure that any urgent referrals are dealt with immediately, in conjunction with child protection partners.

Study data documentation will be produced in accordance with Data Documentation Initiative principles. Questionnaires, topic guides and other data collection instruments will be stored centrally in Rich Text Format. Quantitative and qualitative data will be stored on secure servers at LSHTM, MRC/UVRI, and the UCL Institute of Education, and anonymised data will be made available for sharing via controlled access procedures after 10 years.

### Ethics and child protection

Ethical requirements for the proposed project are complex. We have extensive experience working with this group of adolescents and have established a working referral system in the study district in conjunction with non-governmental organisations and government partners. Participants will be asked about experience of violence which may be severe, and in some cases, they may be at immediate risk of further violence and/or other acute health difficulties. Health, legal, and psychosocial support is available locally with trained Luganda-speaking counsellors. All of our past work has been approved by LSHTM and the Uganda National Council for Science and Technology.

**Fig. 4:**
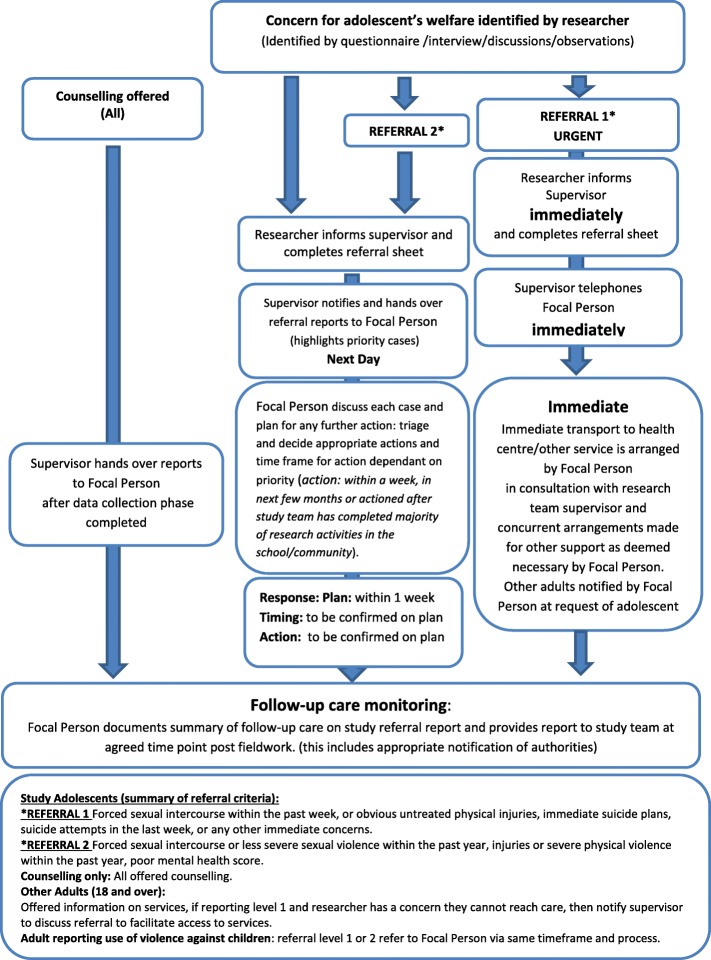
Summary of referral pathways

We have an existing algorithm, developed in conjunction with local service providers and the study team, which dictates how disclosures of abuse from participants will be handled. Adolescents can be referred onwards to a focal person at local child protection partners according to the type, severity and timeframe of violence exposure disclosed (for example, a recent rape would be referred directly to the health centre for post-exposure prophylaxis and emergency contraception, as well as being offered psychosocial support and referral to legal services). Figure 4 describes referral pathways.

The study team continuously monitors the activities of local services providers and the study manager will directly support partner organisations to ensure referrals are responded to in an appropriate and timely manner, as in our past research in this setting [43].

Informed consent can also be a particular issue in longitudinal research, and the team will seek full informed consent at each research contact. Full explanations of the study, and consulting with young people about methods and procedures throughout will serve to enhance their knowledge about and commitment to the research.

### Research governance

The study has an advisory group of academics, practitioners and policy makers who work both in Uganda and internationally. This group offers support, advice, and linkages to broader academic disciplines and communities of practice. We also have a separate ethics advice subcommittee, comprised of experienced researchers and others with specific experience working on research ethics on violence against children. The ethics advice subcommittee provides specific advice on particular cases and issues as they arise. This does not replace either the LSHTM, UVRI or UNCST ethics committees (all of which have approved this research).

### Direct input into intervention development

The third strand of our study takes advantage of our unique and long-standing partnership, between LSHTM, UCL-Institute if Education and Raising Voices, a Ugandan NGO which has a track record of developing and implementing award winning and effective violence prevention interventions [16, 44, 45]. In addition to feeding in to the study instrument design, the data collection planning, and dissemination, Raising Voices intends to directly integrate findings into their ongoing intervention development and programming work. For example, if we find that interaction with non-violent peer groups or staying enrolled in school at Wave 2 is highly protective against partner violence at Wave 3, Raising Voices will develop intervention components designed to specifically improve connections with non-violent peer groups or staying in school to integrate into their existing anti-violence programming in secondary schools. To facilitate component development, we have added costs for intensive workshops for the academic team, Raising Voices and other stakeholders to discuss and digest emerging results after Wave 2 and final results after Wave 3. Raising Voices will also produce a ‘Learning Paper’, which is short technical but non-academic report that aims to provide practical guidance and recommendations for other violence prevention NGOs.

## Discussion

CoVAC will be one of only a handful of prospective cohort studies which include data on violence in adolescence in Africa [1], and will be the only study collecting detailed data on violence where formal longitudinal mediation analysis can be conducted to understand risk and protective factors over adolescence. Specifically, we will be able to examine whether modifiable risk factors such as mental health status, family structure and context, type of violence exposure and school attendance and school climate can mediate associations between early violence exposure and later risk of intimate partner violence and other important health and social outcomes.

For our qualitative study, annual fieldwork will provide detailed insights into how changes in girls’ and boys’ violence-related experiences, perspectives and practices relate to their social contexts, including over the multiple transitions associated with mid-late adolescence, such as between and out of schools, into labour markets, and shifting familial, intimate partner, marital and peer relationships. Four rounds of data collection will help to illuminate contextual pathways that influence later adolescent outcomes, following varying experiences of violence.

The third strand of our study, intervention development, will be directly informed by examination of modifiable risk factors at Wave 2. One of the strengths of the Toolkit is the iterative manner in which it was developed. Research findings over the course of five years will allow us to modify and strengthen the overall intervention to make it more efficient and effective. To our knowledge, this evidence based, iterative approach for intervention development over an extended time period is unique.

### Strengths and limitations

Our study is one of the first of its kind. We will benefit from the strengths of using mixed and complementary research methods, as well as practice-based expertise. We will be able to triangulate emerging findings and ensure that our academic research is generating new knowledge which is directly relevant to programming. Our study is ethically complex, and we are well placed to safely follow up this cohort of adolescents and ensure child protection requirements are met.

#### Attrition

The main risks to the study are around attrition and tracing of participants. Inevitably there will be attrition over time, however levels of attrition are difficult to predict. We have collected a range of contact information for participants which we were able to use to successfully contact them.. We anticipate that there will be higher attrition among some groups, including those who have been exposed to severe violence and those who are not attending school. The original budget includes increased tracing costs for approximately half of the cohort, to account for tracing those who are otherwise more difficult to track.

Collecting qualitative data each year reduces risks of attrition for these participants by providing regular face-to-face contact needed to maintain the research relationships critical to the effectiveness of the study. There is a risk of research fatigue over four rounds of fieldwork, which we will attempt to minimise through the participatory, negotiated data collection methods. The longitudinal qualitative research will drive the discovery of factors related to violence, which enable us to add and refine quantitative measures in subsequent survey waves.

#### Intervention exposure

In our study, almost all participants will have been exposed to the Good School Toolkit, when they were attending primary school. The amount of exposure to the intervention, and also any reductions in violence, vary between participants. Analytically, this can be an advantage, as there will be more variation in experience and use of violence amongst our study participants than there would be in the absence of any intervention. Having more variation, and in particular in the Ugandan context where levels of violence exposure can be extremely high, having some participants with lower exposure levels will actually facilitate statistical analysis.

#### Will child protection responses influence the results?

We are ethically bound to respond to disclosures of violence by participants in our research. We would argue that it is not possible to do longitudinal research on violence without mounting a response to disclosures; and we further maintain that a robust and heavily facilitated response is necessary to ensure participant safety and well-being. However, this does leave open the empirical possibility that child protection responses are in fact influencing the factors under study.

In our research, we offer onward referrals as necessary and counselling support to all participants regardless of what they disclose. In our previous work in Luwero District, roughly 20% of all participants requested support or met child protection criteria to have support provided. For nearly all participants, this comprised one counselling session. Only a very small number of participants (< 10 of more than 3800 interviewed) required any further action such as transport to a health centre, ongoing support, linkage to police or any other service (some additional participants had already sought and received services outside of the study). Analytically, we intend to keep records of who asks for support, what is provided, and to ensure and document any onward referrals that are made. We will be able to examine the characteristics of those who do and do not receive different types of support and where this support happens at Wave 2, examine if this mediates associations between Wave 1 exposures and Wave 3 outcomes.

#### Hawthorne effect

There is also the possibility that repeated participation in research over time may alter factors under study. In our study, participants in the qualitative research will have the most interaction time with the research team. However, repeat interviews over time can be advantageous in qualitative research, as long as their effects are analysed in data interpretation. Participation in research, and particularly research using participatory methods and collecting data over time, can influence the perspectives of participants; for example, generating more critical reflexivity among child participants about violence [3]. Our analysis at each fieldwork period will take account of these influences, and will interrogate the implications of these effects for future interventions.

#### Will results be generalizable?

Our main aim is to try and understand *how* context shapes associations, so the proposed research will generate understanding of how our results may be similar or different to results in other settings and countries. The qualitative research in particular should enable us to interpret our results in the context of geopolitical factors. Our initial (Wave 1) participants were selected to be representative of children attending larger primary schools in Luwero District (schools eligible for our original trial contained 80% of all students in the District; schools and students were randomly selected from all eligible schools to participate in the trial, and 100% of schools approached agreed to participate). In Uganda, 87% of children attend primary school, so our results should be generalisable to much of the population. In Luwero, 27% of population have access to electricity, 79% are living in rural areas, and 92% of children with both parents alive (compared to national estimates of 21, 79, 92% respectively, according to the Bureau of Statistics). Luwero District is also near Kampala, which will allow us to capture the experience of some participants who migrate to Uganda’s largest urban centre. Luwero shares many similarities to other African settings, including economic conditions, rural-urban migration, labour markets, rapid increases in school enrolments particularly at primary level and gendered socio-cultural norms, many of which are undergoing rapid change, as well as persistent norms and practices condoning violence, including commonplace corporal punishment and IPV.

## Implications and conclusion

Violence experienced in adult intimate relationships is tightly linked with experiences of violence in childhood and adolescence, but empirical data which illuminate how these are connected over time are lacking. In Uganda in particular, nearly 60% of adult women have experienced partner violence [46]. This project will generate information on how to identify who might be at risk very early, to inform the best timing of interventions, and on what factors should be targeted in interventions for the Ugandan context. This work will also generate novel data on a host of other early adolescent experiences, early adult outcomes, and how context shapes these relationships. Furthermore, this study aims to shed light on changing social contexts and dynamics that may influence exposure to, experience of and coping with various forms of violence young women and young men experience. Interventions informed by this evidence would have the potential to dramatically reduce the negative health and social consequences of violence and other adverse early experiences on future generations.

## Data Availability

Controlled access to data from CoVAC and the Good Schools Study will be available in 2031 and 2024 respectively.

## Abbreviations

CoVAC: Contexts of Violence in Adolescence Cohort study
IoE: Institute of Education
LSHTM: London School of Hygiene and Tropical Medicine
MRC: Medical Research Counsel
UCL: University of London
UNCST: Uganda National Counsel of Science and Technology
UVRI: Uganda Virus Research Institute

## References

[1] Hillis S (2016). Global prevalence of past-year violence against children: a systematic review and minimum estimates. Pediatrics.

[2] Devries, K., et al., The global prevalence of intimate partner violence*.* Science, 2013. Express online**,** June 20**,** 2013. 10.1126/science.1240937 23788730

[3] Abramsky T (2011). What factors are associated with recent intimate partner violence? Findings from the WHO multi-country study on women's health and domestic violence. BMC Public Health.

[4] Fulu E (2013). Prevalence of and factors associated with male perpetration of intimate partner violence: findings from the UN multi-country cross-sectional study on men and violence in Asia and the Pacific. Lancet Glob Health.

[5] Jewkes R (2013). Prevalence of and factors associated with non-partner rape perpetration: findings from the UN multi-country cross-sectional study on men and violence in Asia and the Pacific. Lancet Glob Health.

[6] Moffitt TE, Caspi A, Findings About Partner Violence From the Dunedin Multidisciplinary Health and Development Study. National Institute of Justice: Research in Brief. NCJ. 1999:170018.

[7] Fang X, Corso PS (2007). Child maltreatment, youth violence, and intimate partner violence: developmental relationships. Am J Prev Med.

[8] Uganda Bureau of Statistics (2016). National Population and Housing Census 2014 - Main Report.

[9] UNESCO. *UNESCO Education Indicators Uganda* 2017 [cited 2019 08/06/2019]; Available from: http://uis.unesco.org/country/UG.

[10] UNDP (2018). Human Development Indices and Indicators.

[11] IHME. *GBD compare: Uganda*. 2017 [cited; Available from: http://www.healthdata.org/uganda.

[12] Ministry of Gender, L.a.S.D. (2015). Violence against Children in Uganda: Findings from a National Survey.

[13] Devries K (2013). School violence, mental health and educational performance in Ugandan primary school children: a cross-sectional survey. Pediatrics.

[14] Devries, K.M., et al., *The Good School Toolkit for reducing physical violence from school staff to primary school students: A cluster-randomised controlled trial in Uganda.* Lancet Global Health, accepted. 10.1016/S2214-109X(15)00060-1 PMC4928210 26087985

[15] World Health Organization (2016). *INSPIRE: Seven Strategies for Ending Violence Against Children*.

[16] Devries KM (2015). The good school toolkit for reducing physical violence from school staff to primary school students: a cluster-randomised controlled trial in Uganda. Lancet Global Health.

[17] Parkes J (2015). Gender violence in poverty contexts: the educational challenge.

[18] Krieger N (2001). Theories for social epidemiology in the 21st century: an ecosocial perspective. Int J Epidemiol.

[19] Parkes J (2013). Conceptualizing gender and violence in research: insights from studies in schools and communities in Kenya, Ghana and Mozambique. Int J Educ Dev.

[20] Anderson K (2005). Theorising gender in intimate partner violence research. Sex Roles.

[21] Bourdieu P, Wacquant L (1992). *An Invitation to Reflexive Sociology*.

[22] Davies B, Harré R (1999). *Positioning and personhood*, in *Positioning Theory*, R.H.a.L.v. Langenhove.

[23] Parkes J (2008). The power of talk: transformative possibilities in researching violence with children. Int J Soc Res Methodol.

[24] Parkes J (2016). Between tradition and modernity: girls’ talk about sexual relationships and violence in Kenya*, Ghana and Mozambique*. Comparative Educ.

[25] (UNCST), U.N.C.f.S.a.T (2014). *National Guidelines for Research involving Humans as Research Participants*.

[26] Madans Jennifer H, Loeb Mitchell E, Altman Barbara M (2011). Measuring disability and monitoring the UN Convention on the Rights of Persons with Disabilities: the work of the Washington Group on Disability Statistics. BMC Public Health.

[27] Joyce HD, Early TJ (2014). The impact of school connectedness and teacher support on depressive symptoms in adolescents: a multilevel analysis. Child Youth Serv Rev.

[28] Blair AH (2017). The alcohol use disorders identification test (AUDIT): exploring the factor structure and cutoff thresholds in a representative post-conflict population in northern Uganda. Alcohol Alcohol.

[29] Knight L (2018). Implementation of the good school toolkit in Uganda: a quantitative process evaluation of a successful violence prevention program. BMC Public Health.

[30] Version, I.-C.T.I.C.A.S.T.C., *ICAST-C: The IPSCAN Child Abuse Screening Tool—Child Version*. 2006, International Society for the Prevention of child abuse and neglect: Aurora, CO.

[31] Garcia-Moreno C (2005). Violence against women. Science.

[32] Wolfe DA (2001). Child maltreatment: risk of adjustment problems and dating violence in adolescence. J Am Acad Child Adolesc Psychiatry.

[33] Antônio Tiago, Hokoda Audrey (2009). Gender Variations in Dating Violence and Positive Conflict Resolution Among Mexican Adolescents. Violence and Victims.

[34] Merrill KG (2018). Effects of a violence prevention intervention in schools and surrounding communities: secondary analysis of a cluster randomised-controlled trial in Uganda. Child Abuse Negl.

[35] Vu L (2017). *Inequitable Gender Norms From Early Adolescence to Young Adulthood in Uganda: Tool Validation and Differences Across Age Groups*. J Adolesc Health.

[36] Goodman R (2001). Psychometric properties of the strengths and difficulties questionnaire. J Am Acad Child Adolesc Psychiatry.

[37] Goodman R (2000). Using the strengths and difficulties questionnaire (SDQ) to screen for child psychiatric disorders in a community sample. Br J Psychiatry.

[38] Johnson JG (2002). The patient health questionnaire for adolescents: validation of an instrument for the assessment of mental disorders among adolescent primary care patients. J Adolesc Health.

[39] Uganda Bureau of Statistics and MEASURE DHS. Uganda DHS Survey 2011. Calverton, Maryland, USA: 2012, Uganda Bureau of Statistics and MEASURE DHS.

[40] Fritz M, Mackinnon D (2007). Required sample size to detect the mediated effect. Psychol Sci.

[41] Heslop J, et al. Making meaning from data on school-related gender-based violence by examining discourse and practice: insights from a mixed methodology study in Ghana and Mozambique. Compare. 2017.

[42] Burke Johnson R (2007). A. Onwuegbuzie, and L. turner, *Towards a definition of mixed methods research*. J Mixed Methods Res.

[43] Devries K (2015). “I never expected that it would happen, coming to ask me such questions” :ethical aspects of asking children about violence in resource poor settings. Trials.

[44] WHO, *INSPIRE: Seven Strategies for Ending violence Against Children*. 2016: Geneva Switzerland.

[45] Abramsky T (2014). Findings from the SASA! Study: a cluster randomized controlled trial to assess the impact of a community mobilization intervention to prevent violence against women and reduce HIV risk in Kampala*, Uganda*. BMC Med.

[46] Uganda Bureau of Statistics - UBOS and ICF (2018). Uganda Demographic and Health Survey 2016.

